# An Evaluation of Personal Cooling Systems for Reducing Thermal Strain Whilst Working in Chemical/Biological Protective Clothing

**DOI:** 10.3389/fphys.2019.00424

**Published:** 2019-04-12

**Authors:** Aaron J. E. Bach, Matthew J. Maley, Geoffrey M. Minett, Stephanie A. Zietek, Kelly L. Stewart, Ian B. Stewart

**Affiliations:** ^1^Institute of Health and Biomedical Innovation, School of Exercise and Nutrition Sciences, Queensland University of Technology, Brisbane, QLD, Australia; ^2^Department of Sport and Exercise Science, University of Portsmouth, Portsmouth, United Kingdom

**Keywords:** heat stress, hyperthermia, microclimate, thermoregulation, occupational, first responder

## Abstract

**Objective:**

The use of personal cooling systems to mitigate heat strain on first-responders achieves two potential performance benefits relative to the absence of such cooling: (1) the completion of a workload with less effort; and/or (2) the completion of a greater workload for the same effort. Currently, claims made by manufacturers regarding the capability of their products for use in conjunction with chemical/biological protective clothing remain largely unsubstantiated. The purpose of this investigation was to evaluate the means by which heat strain can be alleviated during uncompensable heat stress in chemical/biological clothing, using the ASTM F2300-10 methodology.

**Methods:**

Eight healthy males completed five trials of continuous walking (4.5 km h^−1^; 35°C; 49% RH) for up to 120 min while wearing one of four cooling systems and/or a National Fire and Protection Association 1994 Class-3 chemical/biological ensemble. The four cooling methods (ice vest [IV], phase-change vest [PCM], water-perfused suit [WS], and combination ice slurry/ice vest [SLIV]) and no cooling (CON).

**Results:**

We observed significant improvements in trial times for IV (18 ± 10 min), PCM (20 ± 10 min) and SLIV (22 ± 10 min), but no differences for WS (4 ± 7 min). Heart rate, rectal, mean skin, and body temperatures were significantly lower in all cooling conditions relative to control at various matched time points in the first 60 min of exercise. Thermal sensation, comfort and perceived exertion all had significant main effects for condition, and time, there were no differences in their respective interactions.

**Conclusion:**

The IV, PCM, and SLIV produced lower heart rate, mean skin, rectal and mean body temperatures in addition to improved work times compared to control. The WS did not improve work times possibly as a result of the cooling capacity of the suit abating, and magnifying thermal insulation. Considering the added time and resources required to implement combination cooling in the form of ice slurry and ice vest (SLIV), there was no significant additive effect for perception, cardiovascular strain, rectal temperature and total trial time relative to the phase change vest or ice vest alone. This may be a product of a “ceiling” effect for work limit set to 120 min as part of ASTM F2300-10.

## Introduction

Moderate to high-intensity work in the presence of environmental heat stress forces simultaneous demands upon the cardiovascular system by increasing the need for blood flow for thermoregulation and at the active musculature (Kenney et al., 2014). The physiological strain imposed on the individual can be compounded further if workloads are prolonged and heat loss mechanisms are blunted (e.g., encapsulating protective clothing and/or confined spaces) (McLellan et al., 2013; Morrison et al., 2014). Such scenarios can lead to a state of uncompensable heat stress, and if ignored will manifest as signs and symptoms of exertional related heat illness or injury (e.g., heat cramp, heat syncope, and heat stroke) (Bouchama and Knochel, 2002; Casa et al., 2015). A variety of emergency first-responders that require encapsulated personal protection from (potentially) contaminated environments include, but are not limited to, tactical police forces, firefighters, emergency medical technicians, and hazardous materials personnel.

Uncompensable heat stress in these occupations has been demonstrated to significantly limit the operational times before physiological safety limits are reached (Duncan et al., 1979; Faff and Tutak, 1989; Montain et al., 1994). Commercially available personal cooling systems have been proposed as a means to extend these operational times by reducing both cardiovascular and thermal strain (Selkirk et al., 2004; Hostler et al., 2010; Chan et al., 2015; Maley et al., 2018). Put simply, the use of these systems to mitigate heat strain on first-responders achieves two potential performance benefits relative to the absence of such cooling: firstly, the completion of a workload with less effort; and/or secondly, the completion of a greater workload for the same effort. Currently, claims made by manufacturers of personal cooling systems regarding the capability of their products for use in conjunction with chemical/biological protective clothing context remain largely unsubstantiated. The procedure of worker cooling can be divided into three key components, (1) Timing: cooling before, during and/or following work bouts; (2) Application: internal (e.g., ingestion/inhalation) or external cooling (e.g., heat loss at the skin); and (3) Means: passive cooling (exothermic, e.g., absorb body heat and dissipate it into the environment such as an evaporative cooling vest; and/or heat absorbing, utilizing body heat to generate an endothermic reaction such as an ice vest) or active cooling (e.g., uses a power source to circulate a cooling medium, liquid or gas, across the body).

The scientific literature reflects a strong interest in reducing thermal and cardiovascular strain in the first responder and military occupations, suggesting many types of cooling systems are beneficial for workers when used in conjunction with protective clothing (Selkirk et al., 2004; Kenny et al., 2011; Caldwell et al., 2012; House et al., 2013; Glitz et al., 2015; Pryor et al., 2015; Watkins et al., 2018). Though, refinements in technology (e.g., portable pumps, battery size and capacities, synthetic phase change materials) and greater affordability, have seen an ever changing array of commercial systems marketed for sale. Furthermore, few investigations have concurrently assessed multiple types and combinations of cooling systems with chemical/biological clothing in order to identify the most effective system for attenuating the risk of heat illness or injury. Some investigations have shown both passive (Hostler et al., 2010; Kenny et al., 2011; House et al., 2013) and active (Vallerand et al., 1991; Caldwell et al., 2012) cooling systems to be advantageous for workers in protective clothing, with relatively lower cardiovascular and thermal strain leading to improvements in tolerance times and subjective perceptions of exertion and thermal sensation. Even so it remains unclear which systems are superior under circumstances of uncompensable heat stress.

In aggregate, a number of reviews of occupational cooling tend to favor active cooling systems over their passive counterparts (McEntire et al., 2013; Goforth et al., 2014; Mokhtari Yazdi and Sheikhzadeh, 2014; Morrissey and Wang, 2014; Chan et al., 2015). However, these reviews do acknowledge inconsistencies within the literature from which these conclusions are made, due to limitations in current technology (McEntire et al., 2013; Goforth et al., 2014; Morrissey and Wang, 2014), methodological designs (e.g., no control group) (Goforth et al., 2014), real-world practicality (Chan et al., 2015), and the specificity of the cooling application (Mokhtari Yazdi and Sheikhzadeh, 2014). The polarity in findings between similar studies could be attributed to varying methodologies such as ambient environments, the intensity of work prescribed, and the scenarios, timing and frequency in which cooling is applied. In the absence of a uniform evaluation protocol for personal cooling systems, ASTM International developed a human research testing standard, F2300–10 (ASTM International, 2016) in order to: (1) safeguard participant well-being during testing; (2) inform work practices of relevant industrial, first-responder and military occupations; (3) enable the quantification of the effectiveness of various cooling systems; and (4) ensure the conclusions drawn from results are accurate, robust and comparable. This standard provides a clear methodological outline regarding the materials required (e.g., climate chamber, treadmill), participants (e.g., sample size, familiarization), calculations and procedures (e.g., environmental conditions), and as such enables a framework by which comparisons can be made between investigations using different cooling systems.

Due to the paucity of literature comparing personal cooling methods using the standardized F2300-10 framework (ASTM International, 2016), the purpose of this investigation was to distinguish the most effective means by which heat strain can be alleviated during uncompensable heat stress in chemical/biological clothing. Additionally, recent work by our laboratory compared a wide variety of cooling systems during seated rest in the heat. In an environment of 35°C and 50% relative humidity, ice slurry had the greatest effect on rectal temperature (Δ−0.009 ± 0.004°C⋅min^−1^), whilst ice vest influenced mean skin temperature the most (Δ−0.142 ± 0.03°C⋅min^−1^) (Maley et al., 2018). Therefore, we aimed to combine pre-cooling with ice slurry with concurrent cooling of a worn ice vest as a potent and pragmatic solution for encapsulated occupational cooling. It was hypothesized that active cooling systems would be more advantageous to the user than single passive systems (phase change vest and ice vest) and no cooling (control). However, the combination of internal and external passive cooling systems (e.g., ice slurry and ice vest), would provide an additive cooling effect which would see greater improvements in work times before termination criteria were met relative to all other conditions.

## Materials and Methods

### Participants

Following ethical approval from the Queensland University of Technology’s Human Research Ethics Committee, eight healthy males volunteered to participate in this study. The physical characteristics of the recruited sample are displayed in Table 1. All participants were offered verbal and written information concerning the nature and purpose of the study. Participants then completed a health screen questionnaire and provided written consent before commencing the study.

**Table 1:** Participant characteristics (*n* = 8).

Age [y]	Height [cm]	BM [kg]	LM [kg]	FM [kg]	FM [%]	BSA [m^2^]^∗^	VO_2*PEAK*_ [mL⋅kg^−1^⋅min^−1^]
23.6 (3.9)	180 (7)	75.5 (6.4)	64.9 (9.2)	10.3 (4.0)	13.6 (5.2)	1.954 (0.122)	51.6 (4.0)

*Data presented as Mean (SD). BM, body mass; LM, lean mass; FM, fat mass; BSA, body surface area. ^∗^derived from Du Bois and Du Bois (1989).*

### Experimental Design

Participants reported to the laboratory on six occasions. The initial visit consisted of familiarization with the various research equipment (e.g., body temperature sensors), environmental conditions, cooling systems, protective clothing, aerobic capacity testing, and anthropometric measurements. The subsequent five sessions consisted of data collection whereby one of four cooling methods and a no cooling control (CON) were implemented in accordance with F2300-10 (ASTM International, 2016). Inside a controlled climate chamber (4 × 3 × 2.5 m; length, width, height), the environment was maintained at 35.2 ± 0.5°C, 49.2 ± 3.6%, < 1 m⋅s^−1^; conditions were monitored via wet bulb globe thermometer (3M QuestTEMP 36, 3M, United States) approximately positioned at the height of the participants hip. The trials consisted of continuous walking at 4.5 km⋅h^−1^, 1% grade on a calibrated treadmill for up to 120 min while wearing a personal cooling system and/or a National Fire and Protection Association 1994 Class-3 chemical/biological ensemble. During each trial, standard termination criteria were applied as per F2300-10 (ASTM International, 2016): (1) a rectal temperature (T_R_) ≥ 39.0°C; (2) 120 min of walking duration; (3) heart rate equal to 90% of pre-determined maximum; or (4) impending heat illness (e.g., fatigue, nausea, or volitional fatigue). Participants completed a single trial per visit, separated by at least 48 h. Cooling intervention allocation and trial order were randomized (v4 Research Randomizer Form) to ensure a within-participant controlled crossover design.

### Body Composition, Pre-trial Procedures, and Hydration

Body composition was assessed by an accredited Australian and New Zealand Bone and Mineral Society practitioner via dual-energy x-ray absorptiometry (Prodigy, General Electric, United States). Phantom calibration was performed before each scan of the participant lying in the supine position. A single scan lasted approximately 6 min and collected segmental and total body composition of fat mass, lean mass, and bone mineral content. Oxygen consumption via indirect calorimetry (TrueOne 2400, ParvoMedics Inc., United States) was then collected during 8 min of steady-state treadmill walking to ensure the work rate prescribed for all participants (4.5 km⋅h^−1^; 1% grade) fell within the recommendations of the standard (250–400 W) (ASTM International, 2016).

Aerobic capacity and maximal heart rate were derived from a treadmill-based athletic protocol (Hamlin et al., 2012). An initial treadmill gradient of 1% and speed between 8 and 10 km⋅h^−1^ was implemented before 60 s increments of 1 km⋅h^−1^ were applied until the fastest speed the participant could maintain without shifting back on the treadmill. Once this pace was achieved, 1% increases in gradient were applied each 60 s until volitional termination. Breath-by-breath gas analysis was undertaken via indirect calorimetry (TrueOne 2400, ParvoMedics Inc., United States). The gas analysers and pneumotach were calibrated prior to each aerobic capacity test. Participants were fitted with a heart rate monitor and chest strap to allow for calculation of 90% heart rate max, (Team^2^, Polar, Finland).

As per standard procedure (Andersen et al., 1971), in preparation for testing, participants were instructed to abstain from exercise, alcohol, tobacco, caffeinated drinks (i.e., tea, coffee, and energy drinks), vitamin and mineral supplements, in the 24 h preceding any trial. Participants were also asked to consume at least 40 mL⋅kg^−1^ of water the day before the trial (Agostoni et al., 2010) and 500 mL of water 2 h before arrival to the laboratory. Participants arrived at the laboratory for testing in either a morning or afternoon time slot (08:00–09:00 or 13:00–14:00). Each participant was allocated the same testing time for each of their five trials to control for any variance in circadian rhythm and subsequently thermoregulation (Mills, 1966).

Upon arrival, participants were asked to collect a mid-stream urine sample that was assessed for specific gravity (USG – PAL-10S, Atago, Japan). A USG value < 1.020 classified participants as euhydrated (Armstrong et al., 1994). Those with higher values were provided with an additional 500 mL of water to be consumed before a new USG measurement until a reading of ≤ 1.020 was achieved. A single 5 mL venous blood sample was collected from the median cubital vein for the attainment of serum-osmolality using the freezing point depression technique (Osmomat 030, Gonotec, Germany) (Taylor et al., 2012). Following urine and blood collections, nude body mass was measured to the nearest 0.1 kg pre and immediately post trials (Tanita BWB-600, Wedderburn, VIC, Australia), with the participant asked to remove surface sweat with a towel before their post-trial weigh.

### Personal Protective Clothing

The National Fire and Protection Association 1994 Class 3 Extended Response Suit (Lion Apparel, United States) had a total mass of 2.05 kg; consisting of a one-piece fully encapsulating hooded jumpsuit, including outer gloves and booties (1.35 kg) and a face respirator (0.70 kg; Promask – with 2000 PF10 filter, Scott Safety, England). For all trials, per F2300-10 (ASTM International, 2016), participants were instructed to wear (and provided if needed) a standard undergarment consisting of a *t*-shirt, shorts, sports socks, underwear, and athletic shoes. Participants donned all garments within 6 min, then entered the chamber and stood still for 1 min before commencing walking.

### Personal Cooling Systems

The systems chosen consisted of three passive systems and a single active system. All systems were selected based upon their superior performance, compatibility with the required protective clothing for testing and ensuring at least one active and passive system be comparable. One experimental condition paired an extrinsic (ice vest) and intrinsic (ice slurry ingestion) passive cooling system.

#### Cooling Vests

Two different cooling vests were tested: (1) an ice-based cooling vest (IV), stored in a -20°C freezer (ICEEPAK Australia, Australia – 1.2 kg); and (2) a non-ice-based cooling vest with a melting temperature of 14°C (PCM), stored in a 4°C fridge (KewlFit, Model 6626-PEV, TechNiche, United States – 1.8 kg).

#### Water-Perfused Suit (WS)

Participants donned a three-piece portable battery-operated water-perfused suit (BCS4 Cooling System, Med-Eng, Canada) that covered the body, excluding the face, hands, and feet. The WS consisted of tubing sewn into a stretchable pullover, trousers, and hood (Supplementary Figure S1). Water was circulated at ∼375 mL⋅min^−1^ from an integrated portable pump (Delta Wing Pump, Med-Eng, Canada) connected to a 2 L reservoir initially containing 90% ice and 10% water. This resulted in ∼10°C water entering the WS when first turned on (total system mass – 4.7 kg).

#### Ice Slurry + Ice Vest (SLIV)

In the 30 min prior to the walking trial, participants ingested 7.5 g⋅kg^−1^ of ice slurry (2.2% carbohydrate; −2.1 ± 0.3°C) at a rate of 1.25 g⋅kg^−1^ every 5 min to standardize the ingestion rate (Siegel et al., 2010). Each drink was prepared using a slurry machine (Model SSM-180, ICETRO, South Korea) with the same diluted flavoring used for each participant (Fruchilla Natural Lemon, Rainbow Syrup Company Pty Ltd., Australia).

### Body Temperature and Heart Rate

After verification of adequate hydration, participants self-inserted a single-use disposable rectal thermistor (YSI 400, DeRoyal, United States – [ ± 0.1°C]) 12 cm past the anal sphincter to measure T_R_ (International Organisation for Standardisation, 2004). During the trial, the thermistor was connected to an associated wireless logger, worn on a belt inside the protective ensemble, programmed at a data collection frequency of 1 Hz (T-TEC7, Temperature Technology, Australia – [± 0.2°C]). Mean skin temperature (${\bar{\text{T}}}_{\text{SK}}$) was measured with thermocron loggers set at 0.2 Hz (DS1971-F5 iButton^®^, Maxim Integrated, United States – [± 0.5°C; resolution: 0.0625°C]), and placed at four sites in accordance with International Organisation for Standardisation (2004). To ensure adhesion during exercise and minimize the influence of a microenvironment each thermocron logger was held in place with a single piece of adhesive tape (3.8 cm width, Premium Sportstape, Leuko, Germany). Participants were then fitted with a chest strap and heart rate monitor (Team^2^, Polar, Finland).

### Subjective Scales of Temperature and Exertion

A baseline recording of thermal comfort and sensation was collected inside the chamber in the minute immediately prior to walking. Discrete scale measures of thermal sensation, thermal comfort, and rating of perceived exertion (RPE) were subsequently recorded at 15 min intervals and immediately before trial termination. Thermal sensation was assessed using a modified 13 point scale (Gagge et al., 1969) with the following numerical-verbal anchors: 1 “unbearably cold,” 2 “extremely cold,” 3 “very cold,” 4 “cold,” 5 “cool,” 6 “slightly cool,” 7 “neutral,” 8 “slightly warm,” 9 “warm,” 10 “hot,” 11 “very hot,” 12 “extremely hot,” and 13 “unbearably hot.” Thermal comfort was measured using Gagge’s 9-point scale (Gagge et al., 1969) with the following numerical-verbal anchors: 1 “comfortable,” 1.5, 2 “slightly comfortable,” 2.5, 3 “uncomfortable,” 3.5, 4 “very uncomfortable,” 4.5 and 5 “extremely uncomfortable.” RPE was obtained using the 15 point Borg scale (Borg, 1982) with the following numerical-verbal anchors: 6, 7 “very, very light,” 8, 9 “very light,” 10, 11 “fairly light,” 12, 13 “somewhat hard,” 14, 15 “hard,” 16, 17 “very hard,” 18, 19 “very, very hard,” and 20. Standardized instructions of “rate your perception of thermal sensation in the current environment,” “how comfortable are you with the current environment” (Gagge et al., 1969), and “currently, how hard do you feel the work rate is” (Borg, 1982) were provided to participants.

### Data Analyses

Heart rate, T_R_ and ${\bar{\text{T}}}_{\text{sk}}$ was recorded at 0.5 Hz and averaged at 1 min intervals. Weighted ${\bar{\text{T}}}_{\text{sk}}$ was calculated in accordance with the International Organisation for Standardisation (2004):

$${\bar{\text{T}}}_{\text{sk}}=(0.28\cdot {\text{T}}_{\text{neck}})+(0.28\cdot {\text{T}}_{\text{right scapular}})+0.16\cdot {\text{T}}_{\text{left hand}})+(0.28\cdot {\text{T}}_{\text{right shin}})$$

Mean skin and rectal temperatures were used to calculate mean body temperature ${\bar{\text{T}}}_{\text{B}}$, calculated as (Hardy et al., 1938):

$${\bar{\text{T}}}_{\text{B}}=(0.8\cdot {\text{T}}_{\text{R}})+(0.2\cdot {\bar{\text{T}}}_{\text{sk}})$$

### Statistical Analysis

Normality was assessed using descriptive methods (skewness, kurtosis, and outliers) and inferential statistics (Shapiro-Wilk test). The Greenhouse-Geisser correction was applied when Mauchly’s test of sphericity was violated. A one-way repeated measures analysis of variance (ANOVA) was used to confirm participants arrived in a similar physiological state for each testing day, and for termination times and sweat rates between conditions. We then performed a linear mixed effects analysis of the relationship between the conditions (CON, IV, SLIV, PCM, WS) and the physiological (heart rate, T_R_, ${\bar{\text{T}}}_{\text{sk}}$, ${\bar{\text{T}}}_{\text{B}}$) or perceptual (RPE, thermal sensation, and comfort) variable of interest. As the accuracy of the model estimates is disproportionately influenced when participants terminate trials these variables were only modeled on a complete data set (time: 0 to 60 min). As fixed effects, we entered condition, time (with interaction term) into the model. Time was additionally modeled including the polynomial coefficients’ 0.5, 2, and 3 to determine the best fit. As random effects, we had intercepts for participants to account for the correlation between repeated measures on a participant. Model parameters and data are reported as mean (95% confidence interval, lower and upper bound) unless otherwise stated. Visual inspection of residual plots did not reveal any obvious deviations from homoscedasticity or normality. *P*-values were obtained by likelihood ratio tests of the full model with the effect in question against the model without the effect in question. Cohen’s *d* effect sizes between 0 and 60 min were calculated between CON and personal cooling system [Cohen’s *d* = (Mean Difference/(SD_group1_ +SD_group2_)/2)], and interpreted as small (0.2), medium (0.5), or large (0.8) (Cohen, 1988). ANOVAs were statistical analyzed in SPSS (Version 25.0.0.1), Cohens *d* effect sizes in MS Excel (Office 2013), with the linear mixed effect modeling undertaken in the “lme4” package (Bates et al., 2015) in the statistical software package R (Version 3.4.1).

## Results

### Baseline Data

Participants commenced all five trials from a resting physiological baseline, with no significant differences between trials in heart rate (*p* = 0.998), ${\bar{\text{T}}}_{\text{sk}}$ (*p* = 0.208), T_R_ (*p* = 0.466), ${\bar{\text{T}}}_{\text{B}}$ (*p* = 0.432), urine color (*p* = 0.576), urine specific gravity (*p* = 0.878), urine osmolality (*p* = 0.775), serum osmolality (*p* = 0.642), or body mass (*p* = 0.260).

### Termination Times Criteria

**FIGURE 1:**
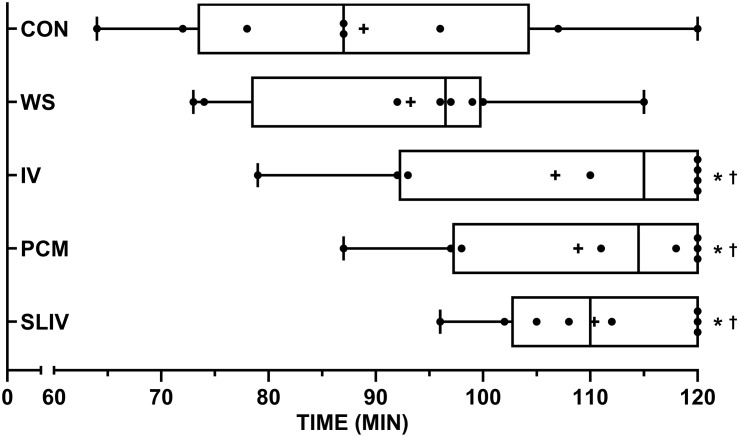
Box and whisker plots for termination times across personal cooling systems (*n* = 8). +, mean; •, individual data points; whiskers, range. ^∗^significantly different (*p* < 0.05) from CON; ^†^ significantly different (*p* < 0.05) from WS. CON, control; IV, ice vest; SLIV, slurry and ice vest; PCM, phase change material; WS, water-perfused suit.

The eight participants successfully completed all 40 trials without volitional fatigue. Termination criteria and times are outlined in Table 2. Significant main effects were observed for cooling method (*p* < 0.001). *Post hoc* analysis revealed significant differences (*p* < 0.05) between termination times for IV, PCM, and SLIV and those of CON and WS (Figure 1). The mean differences (± SD) in trial times between CON and IV were 18 ± 10 min (*p* = 0.012), SLIV (22 ± 10 min, *p* = 0.007), PCM (20 ± 10 min, *p* = 0.006), and WS (4 ± 7 min, *p* = 1.00).

**Table 2:** Termination time (min) and criteria for each cooling condition (*n* = 8).

ID	CON	IV	SLIV	PCM	WS
1	64^*b*^	92^b^	112^b^	97^b^	72^b^
2	71^*a*^	93^a^	108^a^	87^a^	74^a^
3	78^a^	79^a^	105^b^	111^a^	92^a^
4	87^b^	120^c^	102^a^	118^b^	97^a^
5	87^b^	120^c^	96^a^	120^c^	96^a^
6	96^b^	110^b^	120^c^	98^a^	100^b^
7	107^b^	120^c^	120^c^	120^c^	99^b^
8	120^*c*^	120^c^	120^c^	120^c^	115^b^
**Total**	**89 ± 19**	**107 ± 16 ^∗†^**	**110 ± 9 ^∗†^**	**109 ± 13 ^∗†^**	**93 ± 14**

*Note. Superscript is abbreviated termination criteria, ^a^90% heart rate max; ^b^rectal temperature > 39.0°C, ^c^120 min completed. ^∗^significantly different (p < 0.05) from CON; ^†^significantly different (p < 0.05) from WS. CON, control; IV, ice vest; SLIV, slurry and ice vest; PCM, phase change material; WS, water-perfused suit.*

### Physiological and Perceptual Data

**FIGURE 2:**
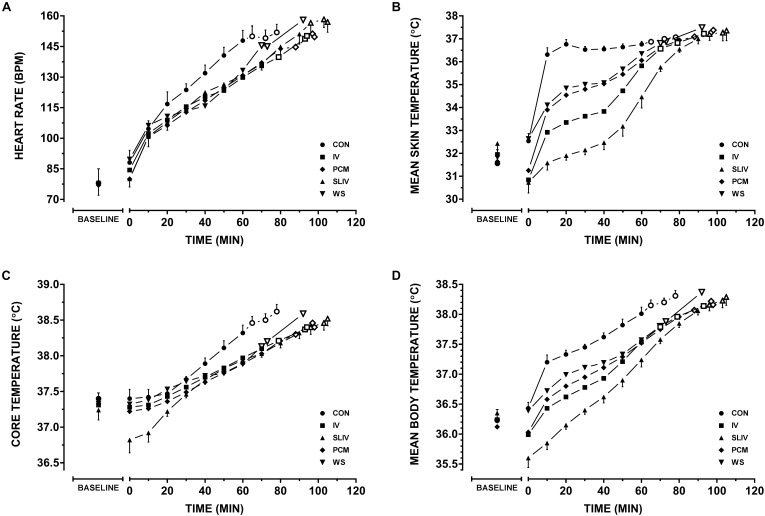
Physiological variables across personal cooling systems. Comparisons between control and all other conditions for **(A)** heart rate, **(B)** mean skin temperature, **(C)** rectal temperature, and **(D)** mean body temperatures during work. Baseline resting data (BASELINE); time walking up to 120 min (Mean ± SD) – for each condition filled markers represent (*n* = 8) participants plotted at 10 min intervals, thereafter, an open marker represent a participant dropping out until *n* = 5. CON, control; IV, ice vest; SLIV, slurry and ice vest; PCM, phase change material; WS, water-perfused suit.

Physiological variables during the trials are displayed in Figure 2. Linear mixed model analysis revealed significant condition, time and interaction effects for all physiological variables (Table 3 presents final model coefficients). The addition of the cooling garments (condition), to the model, lowered T_R_ (χ^2^(4) = 93, *p* < 0.0001) on average by 0.23 ± 0.03°C, ${\bar{\text{T}}}_{\text{SK}}$ (χ^2^(4) = 330, *p* < 0.0001) by 2.21 ± 0.15°C, ${\bar{\text{T}}}_{\text{B}}$(χ^2^(1) = 349, *p* < 0.0001) by 0.62 ± 0.04°C, and heart rate (χ^2^(4) = 63, *p* < 0.0001) by 10 ± 1 b⋅min^−1^. These models were all improved by the addition of an interaction term between time and condition: T_R_ (χ^2^(4) = 52, *p* < 0.0001), ${\bar{\text{T}}}_{\text{SK}}$ (χ^2^(4) = 11; *p* = 0.0220), ${\bar{\text{T}}}_{\text{B}}$ (χ^2^(4) = 22; *p* = 0.0002), and heart rate (χ^2^(4) = 23; *p* = 0.0001). Consequently, *post-hoc* analysis of matched time points between 0 and 60 min, with a Bonferroni adjustment for multiple comparisons, was conducted between CON and each condition for heart rate, T_R_, ${\bar{\text{T}}}_{\text{SK}}$_,_ ${\bar{\text{T}}}_{\text{B}}$ and are displayed in Table 4. Relative to CON, small to large effects were observed over time for heart rate in conditions IV (*d* = 0.2–1), SLIV (*d* = 0.3–1), PCM (*d* = 0.3–0.9), and WS (*d* = -0.1–0.9); large effects for ${\bar{\text{T}}}_{\text{SK}}$ in conditions IV (*d* = 2.7–3.8), SLIV (*d* = 3.2–10.1), PCM (*d* = 1.3–4.6), and WS (*d* = 1.0–3.9); small to large effects for T_R_ in conditions IV (*d* = 0.1–0.9), SLIV (*d* = 0.7–1.8), PCM (*d* = 0.4–1.4), and WS (*d* = -0.2–1.2); and large effects for ${\bar{\text{T}}}_{\text{B}}$ in conditions IV (*d* = 1.5–2.2), SLIV (*d* = 2.3–4.8), PCM (*d* = 1.5–2.3), and WS (*d* = 1.2–1.6).

**Table 3:** Linear mixed model parameter estimates for variables measured during work bouts (*n* = 8).

PARAMETER	HR	${\bar{\text{T}}}_{\text{SK}}$	T_R_	${\bar{\text{T}}}_{\text{B}}$
Intercept	86.90 [75.79, 98.01]^∗∗∗^	33.78 [34.64,35.52]^∗∗∗^	37.32 [37.10, 37.53]^∗∗∗^	36.61 [36.38, 36.84]^∗∗∗^
β, Time	0.39 [0.21, 0.57]^∗∗∗^	−0.02 [−0.04, −0.01]^∗∗^	0.02 [0.02, 0.02]^∗∗∗^	0.01 [0.01, 0.02]^∗∗∗^
β, Time^0.5^	4.76 [3.51, 6.01]^∗∗∗^	0.61 [0.48, 0.74]^∗∗∗^	−0.04 [−0.06, −0.01]^∗^	0.09 [0.06, 0.12]^∗∗∗^
β, Condition				
IV	−1.74 [−6.95, 3.50]	−3.06 [−3.59, −2.53]^∗∗∗^	−0.05 [−0.16, 0.06]	−0.65 [−0.79, −0.52]^∗^
SLIV	−4.95 [−10.16, 0.25]	−3.85 [−4.38, −3.32]^∗∗∗^	−0.46 [−0.57, −0.35]^∗∗∗^	−1.13 [−1.27, −1.00]^∗^
PCM	−5.70 [−10.91, −0.50]^∗^	−2.10 [−2.63, −1.57]^∗∗∗^	−0.10 [−0.21, 0.08]	−0.51 [−0.64, −0.37]^∗^
WS	1.56 [−3.64, 6.77]	−1.35 [−1.88, −0.82]^∗∗∗^	0.05 [−0.06, 0.17]	−0.21 [−0.35, −0.08]^∗^
β, Time × Condition				
IV	−0.27 [−0.42, −0.13]^∗∗∗^	0.02 [0.01, 0.04]^∗∗^	−0.004 [−0.007, −0.001]^∗^	0.0010 [−0.0027, 0.0047]
SLIV	−0.17 [−0.31, −0.03]^∗^	0.00 [−0.01, 0.02]	0.004 [0.000, 0.006]^∗^	0.0042 [0.0004, 0.0079]^∗^
PCM	−0.19 [−0.33, −0.05]^∗^	0.02 [0.00, 0.03]^∗^	−0.005 [−0.008, −0.002]^∗∗^	0.0001 [−0.0036, 0.0038]
WS	−0.34 [−0.49, −0.20]^∗∗∗^	0.01 [−0.01, 0.02]	−0.007 [−0.010, −0.004]^∗∗∗^	−0.0047 [−0.0085, −0.0010]^∗^

*Data presented as mean [95% CI]. Intercept of mixed model denotes control condition alone. β = beta coefficient of model parameter; statistical effect (i.e., the 95% confidence interval does not include zero) indicated by ^∗∗∗^*p* < 0.001; ^∗∗^*p* < 0.01; ^∗^*p* < 0.05. Values are reported to at least one significant decimal place. HR, heart rate;*${\bar{T}}_{\mathrm{SK}}$*, mean skin temperature; T_R_, rectal temperature;*${\bar{\text{T}}}_{\text{B}}$*, mean body temperature; IV, ice vest; SLIV, slurry and ice vest; PCM, phase change material; WS, water-perfused suit.*

**Table 4:** Mixed model analysis of all significant (*p* < 0.05) time points between all conditions, (A) heart rate, (B) mean skin temperature, (C) rectal temperature, and (D) mean body temperatures during work (*n* = 8).

(A)	HR	CON	IV	SLIV	PCM	WS	(B)	${\bar{\text{T}}}_{\text{SK}}$	CON	IV	SLIV	PCM	WS
	CON		50–60	60	50–60	40–60		CON		0–50	0–60	0–50	10–40
	IV	50–60		–	–	–		IV	0–50		10–60	20, 40	0–40
	SLIV	60	–		–	–		SLIV	0–60	10–60		10–60	10–60
	PCM	50–60	–	–		–		PCM	0–50	20, 40	10–60		0
	WS	40–60	–	–	–			WS	10–40	0–40	10–60	0	

**(C)**	**T_R_**	**CON**	**IV**	**SLIV**	**PCM**	**WS**	**(D)**	**${\bar{\text{T}}}_{\text{B}}$**	**CON**	**IV**	**SLIV**	**PCM**	**WS**

	CON		60	0–10, 60	50–60	50–60		CON		0–60	0–60	0–60	50–60
	IV	60		0–10	–	–		IV	0–60		0–30	–	0, 20
	SLIV	0–10, 60	0–10		0–10	0–10		SLIV	0–60	0–30		0–50	0–60
	PCM	50–60	–	0–10		–		PCM	0–60	–	0–50		0
	WS	50–60	–	0–10	–			WS	50–60	0, 20	0–60	0	

*CON, control; IV, ice vest; SLIV, slurry and ice vest; PCM, phase change material; WS, water-perfused suit.*

Only mean differences (± SD) in sweat rate for SLIV were significantly lower (*p* = 0.002) than that of control; CON 9.3 (± 2.8), IV 7.6 (± 4.3), SLIV 6.1 (± 3.0), PCM 6.9 (± 3.2), and WS 8.1 (± 4.9) mL⋅min^−1^. Linear mixed models revealed significant main effects for condition (*p* < 0.0001) and time (*p* < 0.0001) for thermal sensation, comfort and perceived exertion. The addition of condition to the linear mixed models resulted in lower thermal comfort [χ^2^(4) = 43, *p* < 0.0001] by on average 0.61 ± 0.12 au, thermal sensation [χ^2^(4) = 58, *p* < 0.0001] by 1.5 ± 0.25 au, and RPE [χ^2^(4) = 42, *p* < 0.0001] by 1.75 ± 0.33 au. The addition of an interaction effect of condition by time did not improve the model fit for thermal sensation [χ^2^(4) = 4.9, *p* = 0.2997], thermal comfort [χ^2^(4) = 6.3, *p* = 0.1743], nor RPE [χ^2^(4) = 4.0, *p* = 0.4027].

## Discussion

The current investigation evaluated the effectiveness of personal cooling systems at mitigating heat strain during activity while wearing chemical protective clothing. Firstly, in contrast to our initial hypothesis, the active water-perfused suit did not improve work times, cardiovascular, thermal nor perceptual measures compared to the single passive systems (PCM, IV) and control. Secondly, considering the added time and resources required to implement combination cooling in the form of ice slurry and ice vest (SLIV), there was no significant additive effect for perception, cardiovascular strain, T_R_ and total trial time relative to the phase change vest or ice vest alone in work bouts less than 120 min. This may have implications for current practice when combining cooling for first responders during prolonged work in encapsulating clothing.

Often the work intensity, duration and environmental conditions of first responders are dynamic and governed extrinsically, limiting the control of heat illness and injury risk. Personal cooling systems aid in controlling for heat injury risk by significantly reducing the workers thermal and cardiovascular strain. Consider that one (of many) prerequisites to higher work capacities is an ability to extend the time until high deep body temperatures are reached (McLellan et al., 2013; Nybo et al., 2014). That is not to say reductions in skin temperature have not been shown to provide significant benefits for work perception (Chou et al., 2008) and capacity (Smolander et al., 2004). In theory, supplementing ice slurry with an ice vest presents the worker with both internal and external avenues for heat transfer. Mixed method pre-cooling has been investigated in recreational (Cotter et al., 2001; Hasegawa et al., 2006) and team sport athletes (Duffield et al., 2009; Minett et al., 2012). While these methods tend to focus primarily on the cumulative effect of two external cooling interventions, one study by Hasegawa et al. (2006) saw significantly lower thermoregulatory strain by pre-cooling with both water immersion and water ingestion (25°C) than either method on its own. To date, no literature has explored combination (i.e., mixed method) cooling during uncompensable heat stress in chemical/biological clothing. In a recent review, Jay and Morris (2018) presents the notion that cold fluid/ice slurry ingestion during exercise may simply substitute for normal evaporative (e.g., sweat) and dry heat loss (e.g., subcutaneous vasodilation) at the skins surface, and thus provided no net cooling benefit. However, pre-cooling using ice slurry may prevent this from taking place so long as reductions in core body temperature stay within the inter-threshold range (Jay and Morris, 2018). Given that in the case of many first-responders, evaporative heat loss potential is already compromised through diminished convection and elevated water vapor saturation (via sweat) of the encapsulated air, we would hypothesis that the combination of internal and external cooling should be more beneficial than either on their own. We observed significantly lower heart rate, ${\bar{\text{T}}}_{\text{SK}}$, T_R_, and ${\bar{\text{T}}}_{\text{B}}$ for ice slurry/vest, phase change and ice vest relative to control throughout our work protocol in the heat Figure 2. However, we saw no differences in trial times between these passive cooling interventions. One of the limitations of using the F2300-10 (ASTM International, 2016) standard is the potential of a “ceiling” effect for work limits of 120 min making it difficult to delineate the true hierarchy of the three systems as a proportion of participants achieved the maximum duration of 120 min (Figure 1 and Table 2). Therefore, our results likely underestimate performance improvements between each system and these systems relative to control. Though, we did see minimum termination times improve for our participants from ice vest, to phase change vest, to ice slurry/vest (Table 2). It should be noted that typically, occupational guidance is based on these minimum responses rather than average, to ensure that all staff/workers are safe. There is also the potential for the carbohydrate contained in the ice slurry solution to provide further increases in work capacity and/or recovery at low work rates or when work bouts exceed 120 min. Although it was not observed in the current investigation, combination cooling with ice vest with ice slurry may be advantageous when applied between successive work bouts, providing an opportunity to improve thermal recovery, maintain hydration and replenish energy stores.

All four cooling systems implemented in this study had significantly lower cardiovascular and thermoregulatory measures during the first 60 min of walking compared to no cooling. Although, total trial times were significantly less for the perfused water suit relative to the other cooling methods (Figure 1). Notionally, this may be a potential artifact of greater thermal insulation, with eventual sweat saturation of the water suit, the greater metabolic demand due to added bulk and the differences in weight of ∼3 kg (McLellan et al., 2013), and what became the circulation of warm water across the skin due to a single hose covering the entire body (Supplementary Figure S1). As a result there were no differences in trial duration between water-perfused suit and control and subsequently was considered the least effective cooling system in our investigation. This finding is in contrast to a recent meta-analytical review pertaining to concurrent cooling of occupational workers (Chan et al., 2015). Chan et al. (2015) noted a hierarchy of effectiveness (in order of most to least) of air cooled, liquid cooled, hybrid cooling, phase change (including ice) cooling based from 28 investigations. However, the dichotomy between our results and those of Chan et al. (2015) may be due to the reviews exclusion criteria. Investigations were omitted if they used any pre-cooling, reported only physiological outcome measures (e.g., thermal or cardiovascular) in the absence of a performance outcomes (tolerance time, speed, etc.), or if they were conducted in temperatures less than 28°C. This is despite encapsulation during work having the potential to cause heat strain irrespective of temperate, warm or hot ambient temperatures (Montain et al., 1994; Taylor, 2006). Furthermore, no studies included within the review have implemented the ASTM F2300-10 standard protocol (ASTM International, 2016) for evaluating cooling systems.

As the particular water-perfused suit tested in our investigation was considered effective for the first hour of our trials (i.e., where the ice reservoir remained cold), it may still be of interest in operations lasting less than 60 min where chemical/biological protection is used. As there is no one size fits all approach, it is important for key stakeholders to synthesize the relevant resources and literature in order to implement the most pragmatic means of cooling for their workers. With the final decision based upon gauging the occupations specific needs including, the threshold for risk of heat stress (Carter R. et al., 2007), employee characteristics (McGregor et al., 2015; National Institute for Occupational Safety and Health, 2016), expected work durations (National Institute for Occupational Safety and Health, 2016), logistics and preparation time typically available (Bach et al., 2018), allowances for recovery (Carter J.M. et al., 2007), department budgetary constraints (Phuong et al., 2013; Bach et al., 2018), cooling system functionality and integration (Adams et al., 1994; Chan et al., 2013), seasonal and geographic considerations (Carter J.M. et al., 2007; Hanna et al., 2011; McGregor et al., 2015; Nunfam et al., 2018), and the workplace capacity to measure the environment and the worker (McGregor et al., 2015; National Institute for Occupational Safety and Health, 2016).

When interpreting results of heat stress mitigation studies (including our own), readers should remain cognisant that these investigations often lend themselves toward a relatively small sample of unacclimatised, homogeneous, healthy and young male cohort (Kenny et al., 2011; Caldwell et al., 2012; Glitz et al., 2015; Maley et al., 2018). As a consequence the performance benefits described are a “best case scenario” of improvement in safe work duration rather than a reflection of the effectiveness of these cooling apparatus in occupational settings where age, fitness, work experience in the heat and/or acclimatization status are diverse amongst the applicable working population(s) (Gubernot et al., 2014; Cheung et al., 2016).

The ASTM F2300-10 standard protocol recommends the use of 4, 8 or 14 site mean skin temperature measurements via ISO 9886 (International Organisation for Standardisation, 2004), with the 4 site formula explicitly precluded “in conditions close to thermal neutrality and in cold environments… [or] in the case of a highly asymmetrical radiation” (International Organisation for Standardisation, 2004). Likewise, the justification for a 4 site formula for mean skin temperature relies on the assumption of a hot environment, when skin temperature uniformity should be observed, particularly in the case of the user wearing encapsulated clothing (Kenny and Jay, 2011). However, a larger number of measurement sites may be more representative of mean skin temperature, as skin temperature becomes less uniform when cooling systems are placed in direct contact with the skins surface (e.g., ice vests).

## Conclusion

In conclusion, three of the four cooling interventions from this study improved work times during prolonged, uncompensable heat stress in chemical/biological clothing. The ice vest, phase change vest and the combination of ice slurry and ice vest saw lower heart rate, ${\bar{\text{T}}}_{\text{SK}}$, T_R_, and ${\bar{\text{T}}}_{\text{B}}$ and subsequent improvements in work time. Finally, thermal strain was lower for all cooling interventions during the first hour of exercise. However, as the cooling capacity of the water perfused suit abated, potentially due to the sweat saturated garment and ensuing circulation of warmed water across the skin, final termination times did not differ from control.

## Ethics Statement

This study was carried out in accordance with the recommendations of Human Research Ethics Application by QUT’s University Human Research Ethics Committee with written informed consent from all subjects. All subjects gave written informed consent in accordance with the Declaration of Helsinki. The protocol was approved by the QUT’s University Human Research Ethics Committee.

## Author Contributions

AB, MM, GM, and IS conceived and designed the experiments, analyzed the data and wrote the manuscript. AB, MM, SZ, and KS performed the experiments and contributed reagents, materials, and analysis tools.

## Conflict of Interest Statement

The authors declare that the research was conducted in the absence of any commercial or financial relationships that could be construed as a potential conflict of interest.

## Supplementary Material

FIGURE S1 Schematic of piping and three segments of the water-perfused suit, associated pump and reservoir.
