# Supplementary figures and images for: An Evaluation of Personal Cooling Systems for Reducing Thermal Strain Whilst Working in Chemical/Biological Protective Clothing
Source: Front Physiol. 2019 Apr 12;10:424. doi: 10.3389/fphys.2019.00424 (PMC6474400; doi:10.3389/fphys.2019.00424)

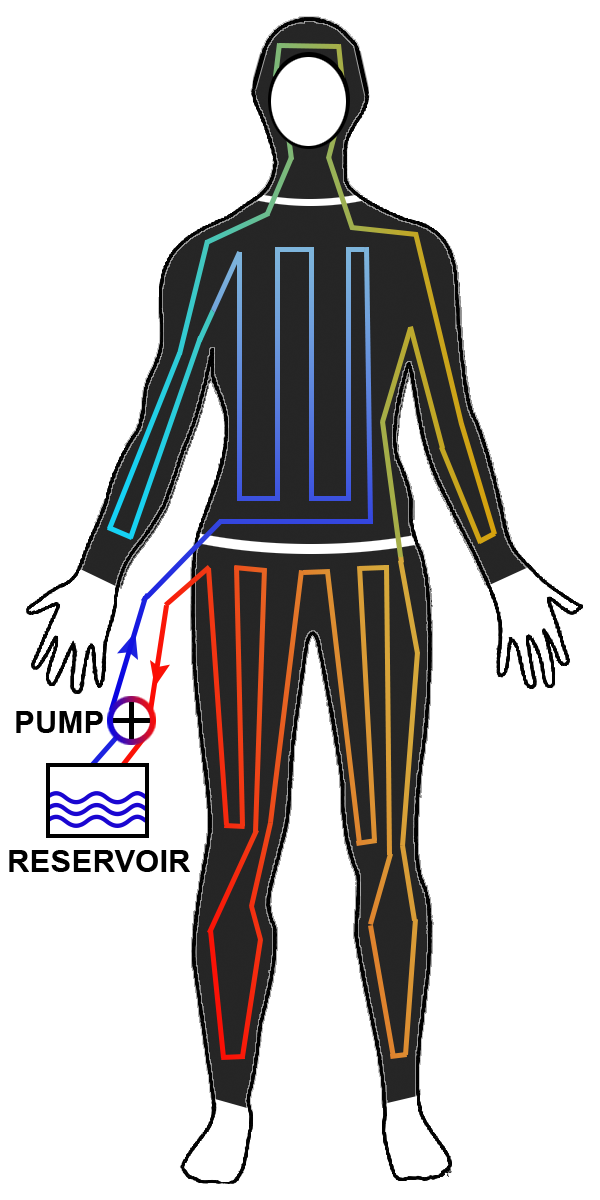

Supplement: FIGURE S1 — Schematic of piping and three segments of the water-perfused suit, associated pump and reservoir. [file Image_1.TIF]
